# Comparative Genomics of *Listeria* Species Recovered from Meat and Food Processing Facilities

**DOI:** 10.1128/spectrum.01189-22

**Published:** 2022-09-06

**Authors:** T. Mafuna, I. Matle, K. Magwedere, R. E. Pierneef, O. N. Reva

**Affiliations:** a Department of Biochemistry, University of Johannesburg, Auckland Park, South Africa; b Bacteriology Division, Agricultural Research Council, Onderstepoort Veterinary Research, Onderstepoort, South Africa; c Directorate of Veterinary Public Health, Department of Agriculture, Land Reform and Rural Development, Pretoria, South Africa; d Biotechnology Platform, Agricultural Research Council, Onderstepoort, South Africa; e Centre for Bioinformatics and Computational Biology, Department of Biochemistry, Genetics and Microbiology, University of Pretoria, Pretoria, South Africa; University of Tennessee

**Keywords:** sequence type, virulence profiles, benzalkonium chloride resistance (BC), stress tolerance, plasmids, prophages, plasmid analysis

## Abstract

*Listeria* species (spp.) are contaminants that can survive in food, on equipment, and on food processing premises if appropriate hygiene measures are not used. Homologous stress tolerance genes, virulence gene clusters such as the *prfA* cluster, and clusters of internalin genes that contribute to the pathogenic potential of the strains can be carried by both pathogenic and nonpathogenic *Listeria* spp. To enhance understanding of the genome evolution of virulence and virulence-associated properties, a comparative genome approach was used to analyze 41 genome sequences belonging to *L. innocua* and *L. welshimeri* isolated from food and food processing facilities. Genetic determinants responsible for disinfectant and stress tolerance were identified, including the efflux cassette *bcrABC* and *Tn6188_qac_1* disinfectant resistance determinant, and stress survival islets. These disinfectant-resistant genes were more frequently found in *L. innocua* (12%) than in *L. welshimeri* (2%). Several isolates representing the presumed nonpathogenic *L. innocua* still carried virulence-associated genes, including *LGI*2, *LGI*3, *LIPI-*3, and *LIPI-*4 which were absent in all *L. welshimeri* isolates. The mobile genetic elements identified were plasmids (*pLGUG1* and *J1776*) and prophages (PHAGE_Lister_vB_LmoS_188, PHAGE_Lister_LP_030_3, PHAGE_Lister_A118, PHAGE_Lister_B054, and PHAGE_Lister_vB_LmoS_293). The results suggest that the presumed nonpathogenic isolates especially *L. innocua* can carry genes relevant to the strain’s virulence and stress tolerance in the food and food processing facilities.

**IMPORTANCE** This study provides genomic insights into the recently expanded genus in order to gain valuable information about the evolution of the virulence and stress tolerance properties of the genus *Listeria* and the distribution of these genetic elements pertinent to the pathogenic potential across *Listeria* spp. and clonal lineages in South Africa (SA).

## INTRODUCTION

The genus *Listeria* consists of 26 spp., of which many of the spp. have been described recently (1, 2). Of these spp., L. monocytogenes and *L. ivanovii* are of primary concern to humans and ruminant animals (3, 4)*. Listeria* spp. can be found ubiquitously in the environment, with *L. innocua* reported as the most isolated *Listeria* spp. (3, 4). L. monocytogenes are regarded as food and food processing environments (FPE) contaminants. Hence, the incidence of listeriosis is mainly linked to the consumption of contaminated foods (5, 6). Contracting L. monocytogenes by immunocompetent individuals tends to cause gastrointestinal symptoms that are transient in nature and often disappear within a short period of time (7). Furthermore, *Listeria* spp. are often used as indicator organisms for environmental sampling and when detected provide a signal that conditions favorable for L. monocytogenes growth or survival could exist (8, 9). Using a broad indicator group, such as *Listeria* spp., increases the chances of finding these niches and controlling them in an effective manner (8, 9).

Of recent, it has been reported that atypical hemolytic *L. innocua* can actively cross the intestinal epithelium and spread systematically to the human liver and spleen. However, it has limited virulent potential compared with virulent strains of L. monocytogenes (10). In addition, in rare cases, the virulent strains of *L. innocua* (11, 12), *L. ivanovii* (13, 14), *L. welshimeri* (15), *L. grayi* (16, 17), and *L. seeligeri* (18) have been reported in human clinical cases, mainly in immunosuppressed individuals (11, 12, 18). The *Listeria* spp. causing infection in humans have been reported to possess L. monocytogenes internalin genes (*inl*A and *inl*B) which encode proteins required for invasion of different cell types as well as incomplete pathogenicity island-1 (*LIPI-*1) comprising of *prf*A, *hly*, and *plc*A genes (12, 19). These strains possess different combinations of L. monocytogenes *LIPI*-1, *inl*A, and *inl*B genes (19). Moura et al. (10) and Rossi et al. (19) investigated the genetic analysis of virulence characters highlighting that food can be a source of potentially pathogenic strains of *Listeria* spp. belonging to spp. generally considered to be innocuous. They reported the L. monocytogenes *inlA* and *hly* virulence determinants can be harbored not only by atypical *L. innocua* strains, but also by *L. welshimeri* and *L. seeligeri* isolates. They concluded that spp. identification is not sufficient to estimate the risk associated with the presence of *Listeria* spp. in food, and both contamination prevention and the identification of contamination sources should be extended to all *Listeria* spp. (10, 19).

Traditionally, foodborne pathogens are characterized using traditional methods such as serotyping and molecular typing assays (9). However, whole-genome sequencing (WGS) has recently emerged as a powerful tool for bacterial characterization and investigation of outbreaks caused by foodborne pathogens including *Listeria* spp. (20–22). WGS-based studies querying the presumed nonpathogenic *Listeria* spp. strains isolated from food and FPE sources in South Africa (SA) are limited. Furthermore, little is known regarding their virulent potent and which sequence types (STs) are circulating among animals, food, and the food processing industry in the country. Hence, genomic insights into the recently recognized expanded diversity of the genus *Listeria* are necessary to improve our understanding of the evolution of virulence and virulence-associated properties of these potentially dangerous pathogens. This knowledge will also enhance the ability to develop and implement testing and food control procedures for the pathogenic and the presumed nonpathogenic *Listeria* spp. This study aimed to institute a comparison study in order to extend our understanding of the phylogenetic relatedness, stress resistance genes, virulence factors, and CRISPR-cas systems from the accessory genome of the presumed nonpathogenic *Listeria* spp. (*L. innocua and L. welshimeri*) isolated in SA compared with pathogenic reference L. monocytogenes strains and including the L. monocytogenes from our previous study (23).

## RESULTS

### General and specific genomic features of the Listeria species genomes isolated from food and food processing environments.

**FIG 1:**
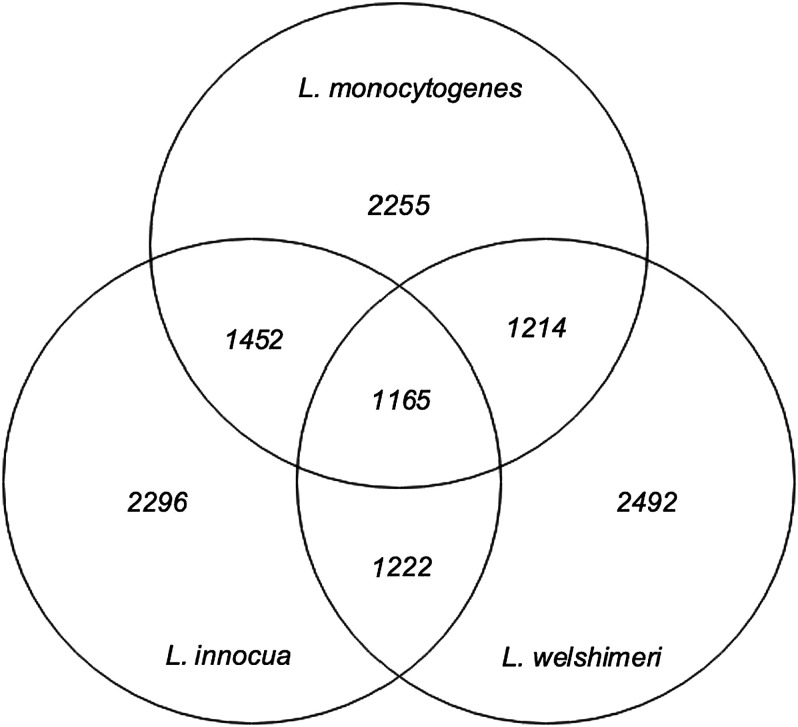
Pangenome analysis of the *Listeria* spp. isolates included in the current study. Grouped by spp. (L. monocytogenes, *L. innocua*, and *L. welshimeri*). Numbers represent gene coding loci associated with one or more species.

**FIG 2:**
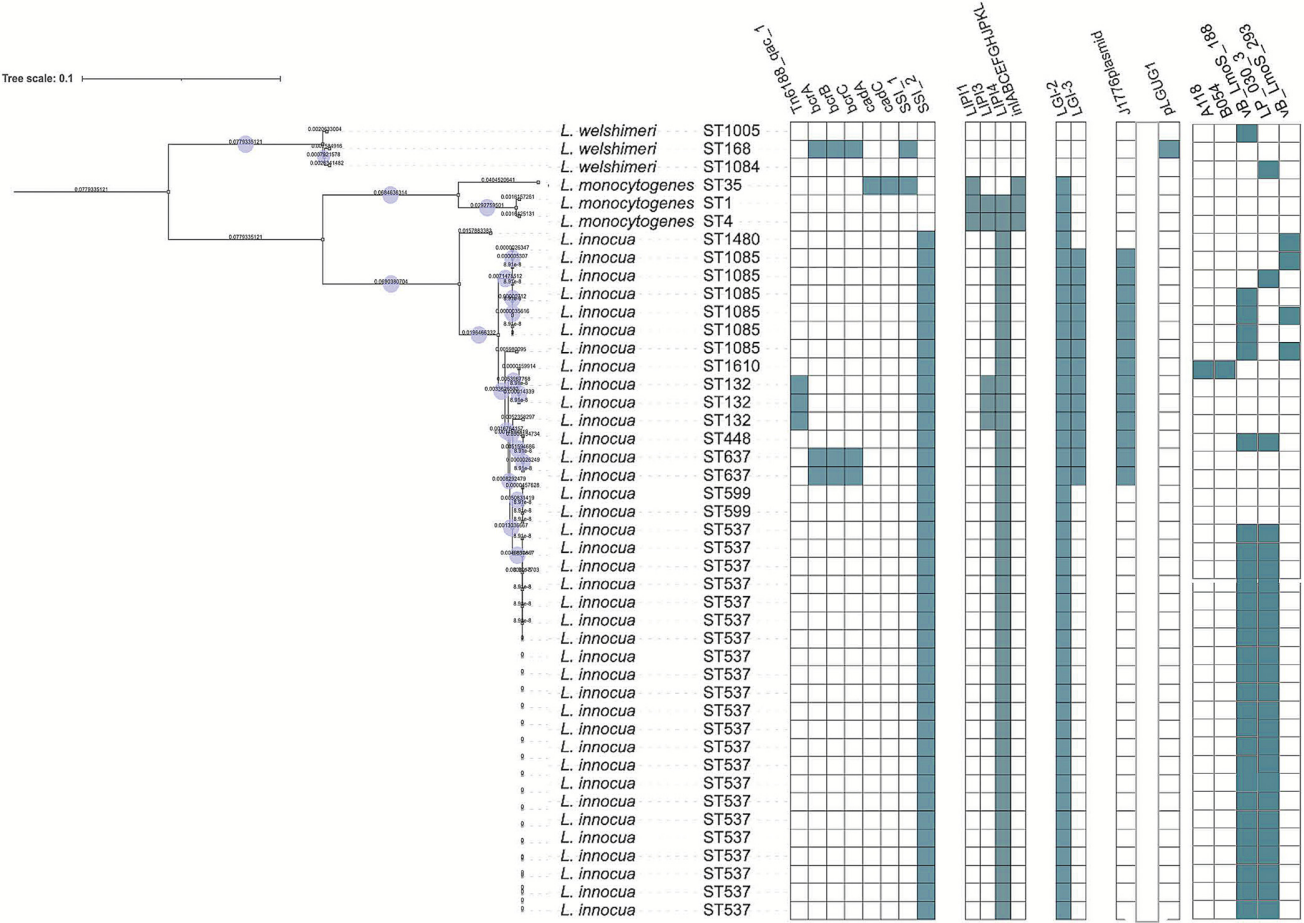
Presence of selected stress tolerance, virulence genes, genomic islands, and plasmids among *Listeria* spp. isolates in this study. The branch length represents the evolutionary time between two nodes. The heatmap to the right of the phylogeny denotes whether the Benzalkonium chloride resistance genes, virulence factors, SSI, plasmid replicons, and prophages were present or absent in the *Listeria* genomes. The presence is indicated by blue and absence is indicated by white. The phylogeny was constructed and annotated using IQ-TREE and visualize by iToL.

An overview of the genetic subtypes and genome characteristics of the isolates in this study is presented in Table 1. The L. monocytogenes EDG-e, CLIP80459, and F2365 genomes ranged from 2.91 to 2.94 Mbp. The *L. innocua* genomes ranged from 2.79 to 3.032 Mbp. Finally, the *L. welshimeri* genome from 2.78 to 2.86 Mbp. The GC content was lowest among *L*. *welshimeri* (36.22% to 36.33%), followed by *L. innocua* (37.26% to 37.44%), and finally L. monocytogenes (37.98% to 38.06%). The total pangenome size between the three *Listeria* spp. contained 11,782 genes across the 44 *Listeria* isolates. The partitioning of genes across the pan-genome was as follows: core, 1,165; softcore, 87; shell, 1,869; and cloud, 8,661 genes. The *L. innocua* pangenome consisted of 9,346 genes, of which 2,296 genes were core to all strains. The *L. welshimeri* pangenome had 3,234 genes, and 2,492 genes were core to all strains. The L. monocytogenes pangenome had 3,481 genes, and 2,255 genes were core to all strains. Genome comparison is summarized in a Venn diagram (Fig. 1) and a core genome phylogenetic tree (Fig. 2).

**TABLE 1:** General and specific genomic features of the *Listeria* spp. genomes isolated from food and processing environments

Isolates	Sequence types (ST)	Species	Genome size (mbp)	No. of contigs	GC (%)	Sample type	Meat category	Establisment category
L0171	ST132	*L. innocua* ^*a*^	2.98	14	37.30	Beef patties	Processed meat-beef	Retail
L1034	ST1005	*L. welshimeri* ^*a*^	2.81	21	36.22	Russian wors chicken	Processed meat-poultry	Retail
L1036	ST1085	*L. innocua* ^*a*^	2.88	13	37.34	beef wors	Processed meat-beef	Retail
L11	ST537	*L. innocua* ^*a*^	2.88	33	37.46	Vienna	RTE-pork	Abattoir
L1221	ST1610	*L. innocua* ^*a*^	3.03	42	37.29	Chicken leg Quarter	Raw-poultry	Cold store
L13	ST537	*L. innocua* ^*a*^	2.88	24	37.47	Vienna	RTE-poultry	Processing plant
L1335	ST1480	*L. innocua* ^*a*^	2.88	15	37.26	Chicken wing	Raw-poultry	Retail
L14	ST537	*L. innocua* ^*a*^	2.88	17	37.47	Polony	RTE-poultry	Processing plant
L145	ST637	*L. innocua* ^*a*^	2.90	17	37.46	Minced meat	Processed meat-beef	Butchery
L15	ST537	*L. innocua* ^*a*^	2.88	18	37.47	Polony	RTE-poultry	Processing plant
L166	ST1085	*L. innocua* ^*a*^	2.88	13	37.34	Beef patties	Processed meat-beef	Butchery
L18	ST537	*L. innocua* ^*a*^	2.88	19	37.47	Wors	Processed meat-beef	Retail
L181	ST1085	*L. innocua* ^*a*^	2.88	15	37.33	Beef wors	Processed meat-beef	Retail
L186	ST1085	*L. innocua* ^*a*^	2.88	15	37.33	Beef mince meat	Processed meat-beef	Retail
L19	ST537	*L. innocua* ^*a*^	2.88	20	37.47	Mince	Processed meat-beef	Retail
L21	ST537	*L. innocua* ^*a*^	2.88	15	37.47	Wors	Processed meat-beef	Retail
L22	ST537	*L. innocua* ^*a*^	2.88	19	37.47	Wors	Processed meat-beef	Retail
L23	ST1084	*L. welshimeri* ^*a*^	2.78	17	36.26	Mince	Processed meat-beef	Retail
L24	ST537	*L. innocua* ^*a*^	2.88	16	37.47	Beef minced meat	Processed meat-beef	Butchery
L241	ST599	*L. innocua* ^*a*^	2.79	14	37.42	Pork Russian	Processed meat-pork	Butchery
L3	ST537	*L. innocua* ^*a*^	2.88	17	37.47	Patties	Processed meat-pork	Retail
L4	ST537	*L. innocua* ^*a*^	2.88	18	37.47	Vienna	RTE-pork	Processing plant
L505	ST1085	*L. innocua* ^*a*^	2.92	52	37.31	Pork Russian	Processed meat-pork	Butchery
L519	ST1085	*L. innocua* ^*a*^	2.88	17	37.33	Beef mince	Processed meat-beef	Retail
L52	ST448	*L. innocua* ^*a*^	2.87	15	37.37	Wors	Processed meat-beef	Butchery
L57	ST537	*L. innocua* ^*a*^	2.88	17	37.47	Mince	Processed meat-beef	Retail
L59	ST537	*L. innocua* ^*a*^	2.88	16	37.48	Patties	Processed meat-beef	Retail
L6	ST537	*L. innocua* ^*a*^	2.89	25	37.48	Polony	RTE-pork	Processing plant
L60	ST537	*L. innocua* ^*a*^	2.88	18	37.48	Beef biltong	RTE-Beef	Retail
L61	ST537	*L. innocua* ^*a*^	2.88	16	37.47	Beef biltong	RTE-beef	Retail
L62	ST168	*L. welshimeri* ^*a*^	2.86	18	36.33	Wors	Processed meat-beef	Retail
L63	ST537	*L. innocua* ^*a*^	2.88	22	37.47	Mince	Processed meat-beef	Retail
L64	ST537	*L. innocua* ^*a*^	2.88	17	37.47	Beef biltong	RTE-beef	Retail
L69	ST537	*L. innocua* ^*a*^	2.88	16	37.47	Patties	Processed meat-beef	Retail
L7	ST537	*L. innocua* ^*a*^	2.88	17	37.46	Patties	Processed meat-beef	Processing plant
L735	ST599	*L. innocua* ^*a*^	2.79	172	37.41	Beef mince	Processed meat-beef	Retail
L755	ST637	*L. innocua* ^*a*^	2.88	79	37.46	Beef mince	Processed meat-beef	Butchery
L8	ST537	*L. innocua* ^*a*^	2.88	18	37.47	Mince	Processed meat-beef	Retail
L80	ST537	*L. innocua* ^*a*^	2.88	16	37.47	Beef biltong	RTE-beef	Butchery
L84	ST132	*L. innocua* ^*a*^	2.96	25	37.30	Beef patties	Processed meat-beef	Butchery
L85	ST132	*L. innocua* ^*a*^	2.96	27	37.30	Minced meat	Processed meat-beef	Butchery
EDG-e	ST35	L. monocytogenes ^*b*^	2.94	1	37.98	N/A	N/A	N/A
Str.CLIP80459	ST4	L. monocytogenes ^*b*^	2.91	1	38.06	N/A	N/A	N/A
Str.F2365	ST1	L. monocytogenes ^*b*^	2.91	1	38.04	N/A	N/A	N/A

a Nonpathogenic strains.

b Pathogenic strains.

The most common ST identified in *L. innocua* strains were ST537 (*n* = 22, 56%) followed by ST1085 (*n* = 6, 14.6%). The STs found in the *L*. *welshimeri* strains were ST1005, ST1084, and ST168 (Table 1; Fig. 2).

### Phylogenetic analysis of the presumed nonpathogenic Listeria species.

The phylogenetic analysis, based on 44 *Listeria* spp. core genes showed that L. monocytogenes (EDG-e, Str.CLIP80459, and Str.F2365) were closely related and shared a more significant (*P* < 0.05) number of genes mainly with *L. innocua* (*n* = 1,452) in comparison with the number of genes shared with *L. welshimeri* (*n* = 1,214) (Fig. 1). The number of genes shared exclusively between *L. innocua* and *L. welshimeri* was 1,222 genes from the pangenome. The phylogenetic analysis showed that *L. welshimeri* cluster was distinct from L. monocytogenes and *L. innocua* clusters, as displayed in Fig. 2. Within the *L. innocua* clade, the diversity within the strain in this clade is based on the presence or absence of orthologous genes (Fig. 2).

### Stress resistance determinants in the presumed nonpathogenic Listeria species.

The plasmid-borne benzalkonium chloride (BC) resistance *bcrABC* cassette and *Tn6188_qac_1* were the two disinfectant resistance determinants identified among the study isolates. The *bcrABC* cassette was only present in ST637 belonging to *L. innocua* and ST168 belonging to *L. welshimeri* (Fig. 2). The *Tn6188_qac_1* gene was only present in *L. innocua* harboring ST132. The *cadA* and *cadC* genes that confer resistance to cadmium were only identified in L. monocytogenes belonging to ST35 (Fig. 2).

*Listeria* Genomic island 2 (*LGI2*), which encodes a large arsenic resistance operon (*arsD1A1R1D2R2A2B1B2*) was present in all the *L. innocua* isolates in the present study and showed 100% identity with *LGI2* recovered from https://bigsdb.pasteur.fr/listeria/ and NCBI databases. The *Listeria* genomic island 3 (*LGI3*) was identified in *L. innocua* belonging to ST1085, ST132, ST637, and ST448 (Fig. 2). The *LGI*2-3 were only identified in *L. innocua* strains that harbored other resistance genes, and none of these islands were found in *L. welshimeri* (Fig. 2). The L. monocytogenes stress survival islets (SSI-1 and SSI-2) that encode resistance to stress conditions such as temperature, pH, and osmotic stress were identified in the present study. The SSI-2 was identified in all *L. innocua* isolates analyzed, whereas SSI-1 was only present in 1 isolate belonging to *L. welshimeri* ST168 (Fig. 2).

### Virulence determinants in the presumed nonpathogenic Listeria species.

The distribution of essential virulence *determinants* in the genus *Listeria* is shown in Fig. 2. *Listeria* pathogenicity islands *LIPI*-1 to *LIPI*-4, which contributes to pathogenesis and increase the bacteria’s virulence, were investigated. The *LIPI*-1, *LIPI*-3, and *LIPI*-4 were identified in the pathogenic *Listeria* spp. (EDG-e, Str.CLIP80459, and Str.F2365) whereas complete *LIPI*-1 was not detected in *L. innocua* and *L. welshimeri* isolates (Fig. 2). The complete sequences of *LIPI*-3 consisting of eight genes (*IIsA*, *IIsB*, *IIsD*, *IIsG*, *IIsH*, *IIsP*, *IIsX* and *IIsY*) was found in all *L. innocua* ST132 isolates (Fig. 2). The complete sequences of *LIPI*-4 consisting of five genes (LM9005581_70009 to LM9005581_70014) were found in all *L. innocua* isolates, but this pathogenic island was not found in any *L. welshimeri* isolates (Fig. 2). Genes of internalin synthesis, including *inlABCEFJK*, were found exclusively in the L. monocytogenes genomes (Fig. 2).

### Mobile genetic elements in the presumed nonpathogenic Listeria species.

Two plasmids (*pLGUG1* and J1776) were identified in the present isolates and showed more than 95% identity and coverage to their corresponding plasmids from PLSDB (24) (Fig. 2). The plasmid found in *L. welshimeri* was *pLGUG1*. The J1776 plasmid was only present in *L. innocua* isolates belonging to ST1085, ST132, ST637, ST1610, and ST448. A total of five intact prophages were also identified in the present isolates and showed 95% identity and coverage against the PHASTER server reference sequences (25) (Fig. 2). The most common prophages identified in the present isolates were PHAGE_Lister_vB_LmoS_188 and PHAGE_Lister_LP_030_3, and both prophages were detected in all *L. innocua* ST537 isolates. The prophages detected in *L. welshimeri* were PHAGE_Lister_vB_LmoS_188 in ST1005 and PHAGE_Lister_LP_030_3 in ST1084, respectively. Other known prophages identified were PHAGE_Lister_A118, PHAGE_Lister_B054, and PHAGE_Lister_vB_LmoS_293 detected in *L. innocua* belonging to ST448, ST132, ST1610, and ST1480 isolates (Fig. 2). The plasmids and prophages identified in the presumed nonpathogenic strains were absent in L. monocytogenes (EDG-e, Str.CLIP80459, and Str.F2365).

### The presence of CRISPR-cas systems in the presumed nonpathogenic Listeria species.

The presence of CRISPR-cas systems (a system that degrades foreign genetic elements) in *Listeria* spp. were investigated. A total of three CRISPR-cas system types, including 12 different cas genes, were present in the study isolates (Table 2). The CAS-Type IIA system was present in 11 *L. innocua* strains belonging to ST537, ST637, ST132, ST599, and ST1610. The CAS-Type IIA system in *L. innocua* strains comprises of the following cas genes: Cas3_0_I, Cas3_1_I, Cas9_1_II, Cas1_0_II, Cas2_0_I-II-III, and Csn2_0_IIA (Table 2). The CAS-Type IB system was present in 15 *L. innocua* strains with cas genes Cas2_0_I-II-III-V, Cas3_0_I, Cas5_0_IB, Cas7_1_IB, Cas8a1_0_IB, Cas6_0_I-III, Cas5_0_IB, and Cas10_1_IIIB belonging to ST132, ST448, ST637, ST1085, ST637, and ST1610 (Table 2). The CAS-Type IIC system (Cas9_1_II, Cas1_0_II, Csn2_0_IIA) was only present in 1 *L. innocua* strain belonging to ST 1610. *L. welshimeri* ST1005 was found with only CAS-Type IIA system consisting of cas genes Cas2_0_I-II-III-V, Cas3_0_I, Cas5_0_IB, Cas7_1_IB, Cas8a1_0_IB, and Cas6_0_I-III (Table 2).

**TABLE 2:** Different types of CRISPR-cas systems types and Cas genes detected in *Listeria* spp. genomes^*a*^

Types	Cas genes	Isolates	Sequence types (ST)
CAS-type IIA	*Cas3_0_I, Cas3_1_I, Cas9_1_II, Cas1_0_II, Cas2_0_I-II-III, Csn2_0_IIA*	*ST537*	*L. innocua*
	*Cas9_1_II, Csn2_0_IIA*	*ST637, ST132, ST599, ST1610*	
CAS-type IB	*Cas2_0_I-II-III-V, Cas3_0_I, Cas5_0_IB, Cas7_1_IB, Cas8a1_0_IB, Cas6_0_I-III, Cas5_0_IB*	*ST132*	*L. innocua*
	*Cas10_1_IIIB*	*ST448, ST637, ST1085, ST637, ST1610*	
CAS-type IIC	*Cas9_1_II, Cas1_0_II, Csn2_0_IIA*	*ST1610*	*L. innocua*
-	Cas3_0_I, Cas3_1_I	*ST637*	*L. innocua*
-	Cas2_0_I-II-III	*ST1610*	*L. innocua*
CAS-type IIA	Cas2_0_I-II-III-V, Cas3_0_I, Cas5_0_IB, Cas7_1_IB, Cas8a1_0_IB, Cas6_0_I-III	*ST1005*	*L. welshimeri*
CAS-type IIA	-	*ST35*	EDG-e^*b*^
-	-	*ST4*	Str.CLIP80459^*b*^
-	-	*ST1*	Str.F2365^*b*^

a -, not defined.

b L. monocytogenes.

### Comparative genomic analysis of resistance genes in the presumed nonpathogenic species and Listeria monocytogenes strains isolated from SA.

The current presumed nonpathogenic isolates were compared with our previously published by (26, 27) pathogenic L. monocytogenes, isolated between 2014 and 2019 from food and FPEs. Benzalkonium chloride resistance markers, including the plasmid-borne BC resistance *bcrABC* cassette and *Tn6188_qac_1* detected in the present study (Fig. 2) were also detected in our L. monocytogenes genomes from the previous study (26) except for the *Tn6188_qac_1.* For SSI markers, SSI-1 which was observed only in one isolate belonging to *L. welshimeri* in the present study (Fig. 2), was also observed in the L. monocytogenes (*n* = 86, 55%) from our previous study (26). However, the SSI-2 which was observed in all the present *L. innocua* isolates (Fig. 2), was only observed in (*n* = 11, 7.7%) of the L. monocytogenes (26). For the virulence genes, the *LIPI-*1 which was observed in (*n* = 16, 7.4%) of the L. monocytogenes isolates (27) was absent in all the presumed nonpathogenic strains in the present study. The internalin gene family members, including *inlABCEFJK* which were present in 90% of the L. monocytogenes (26) isolates were absent in all the present presumed nonpathogenic strains. However, *LIPI-*3 and *LIPI-*4 which were observed in the present *L. innocua* strains (Fig. 2), were also observed in (*n* = 47, 21.7%) and (*n* = 4, 1.8%), respectively, in our previous L. monocytogenes (27). The plasmids and prophages identified in the presumed *nonpathogenic* strains were also observed in L. monocytogenes strains (26) except for the *pLGUG1* plasmid.

## DISCUSSION

This study presents the intrinsic genetic attributes of pathogenic and presumed nonpathogenic *Listeria* spp. (*L. innocua* and *L. welshimeri*) isolated from FPE in SA through characterization of virulence factors, stress tolerance genes, and phylogenetic relationships between the isolates. In the present study, a total of eight and three unique STs belonging to *L. innocua* and *L. welshimeri* were detected in the selected isolates, respectively. The most common STs in the *L. innocua* were ST537, ST1085, and ST132. The STs identified in *L. welshimeri* were ST1005, ST1084, and ST168. This MLST results tally with two studies conducted in China (28) and Ireland (6) which showed the STs detected in the presumed nonpathogenic *Listeria* spp. were ST537, ST1005, ST1084, and ST168 belonging to *L. innocua* and *L. welshimeri,* respectively. The pangenome and phylogenetic analysis revealed that L. monocytogenes share more genes mainly with *L. innocua* (*n *= 1,452), than with *L*. *welshimeri* (*n *= 1,214). This is supported by the likelihood of this spp. being closer to each other in evolutionary terms (8). Similar findings were observed by den Bakker et al. (3) and Palaiodimou et al. (6) Listeria innocua is a nonhemolytic *Listeria* spp. found in similar environments common to L. monocytogenes, and the high number of genes shared between the two spp. compared with *L. welshimeri* could have been a result of horizontal gene transfer (29).

The ability of *Listeria* strains to adapt and survive in food and FPE is based on the development of resistance to disinfectant (QACs) such as BCs as well as biofilm production. For these bacteria to further thrive in the environment, they must tolerate many stressful conditions that are unfavorable to their survival (6, 25, 26, 30). In fact, previous studies have suggested disinfectant resistance markers such as the *bcrABC* cassette, *emrC*, *emrE*, *qacA*, *qacC*, *qacH*, and *qacEΔ1* determinants have been previously identified in *Listeria* spp. These markers are associated with resistance to QACs, a class of disinfectant used in the food processing facility (6, 26, 31). The disinfectant resistance determinants such as bcrABC cassette were identified in ST637, ST168 belonging to *L. innocua* and *L. welshimeri*, respectively, and *Tn6188_qac_1* in *L. innocua* harboring ST132 in the present study. These genes were also detected in our L. monocytogenes from our previous study (26). These known disinfectant-resistant determinants point to an associated survival and persistent contamination dynamics in the food and FPEs by these strains (ST637, ST132, and ST168) (6, 8, 29). Additionally, none of the heavy metal resistance markers (cadmium and arsenic) which provide resistance to stress conditions were observed in the present presumed nonpathogenic *Listeria* spp. isolates.

Previous studies revealed genomic islands could contain genes that improve the fitness of a strain (23, 32). The presence of these islands might lead to increased FPEs survival and pathogenic potential of the strain. Of the genomic islands identified in *Listeria* spp., *LGI1* and *LGI3* have been associated with survival and persistence in food and FPEs (23, 32), and *LGI2* potentially provides an increased survival, persistence, and virulence potential (32–34). *LGI2* was identified in all the *L. innocua* isolates, while *LGI3* was identified in *L. innocua* isolates belonging to ST1085, ST132, ST637, and ST448. The islands can potentially cause increased virulence and environmental fitness within FPEs. These isolates can maintain the arsenic and cadmium resistant determinants along with various metabolism, transport, stress resistance, transposon, and regulatory genes (32).

The SSI-1, which has been linked to tolerance toward acidic, bile, gastric, and salt stresses (35–37), was identified in a single isolate belonging to *L. welshimeri* ST168 and this SSI was also identified in 55% of our previously published L. monocytogenes isolates (26). The SSI-2, responsible for survival under alkaline and oxidative stresses, was observed in all *L. innocua* isolates analyzed. The L. monocytogenes harbored 7.7% of the SSI-2 (32) and none of SSI-2 was found in *L. welshimeri*. Palaiodimou et al. (6) reported similar findings exhibiting *L. welshimeri* and L. monocytogenes harbored SSI-1 compared with *L. innocua* strains, which carried SSI-2 only. The presence of SSI-2 indicates an increased ability of *L. innocua* strains to survive under alkaline and oxidative stresses in FPEs (6, 36).

The expression of the most key *Listeria* spp. virulence factors identified to date is under the control of the *prfA* virulence cluster or the *Listeria* pathogenicity island (*LIPI*) which encodes several essential proteins for intracellular survival and motility (3, 37). The *LIPI*-1 (*Prf-A* dependent virulence cluster genes that are critical in the infectious cycle of L. monocytogenes) and *LIPI*-2 (the former encoding the primary virulence gene locus in L. monocytogenes and the latter encoding virulence factors in *L. ivanovii*) were not detected in the current isolates. The *LIPI*-3 pathogenicity island encodes *Listeriolysin S* and is associated with increased L. monocytogenes virulence capabilities ubiquitous in ST132 belonging to *L. innocua* and was also detected in 21% of our previous L. monocytogenes (27). The *LIPI*-4 pathogenicity island, which is associated with L. monocytogenes hype-virulence by enhancing the invasion of the central nervous system and placenta, was detected in all the *L. innocua* isolates in the present study and was also found in 1.8% of the L. monocytogenes from our previous study (27). None of the *L. welshimeri* strains harbored *LIPI*-1 to *LIPI*-4 found in other *Listeria* spp. The findings correspond with the results of a study conducted in Ireland that did not detect any of the *LIPI* in *L. welshimeri* (6). However, the same study did not detect *LIPI-*3 in their study isolates but this genomic island was detected in the present *L. innocua* isolates and in L. monocytogenes. A study by Matle et al. (27) reported *LIPI-*4 was identified in all their *L. innocua* studied. The internalin gene family members (*inlABCEFJK*) were not detected in any presumed nonpathogenic *Listeria* strains analyzed in the present isolates. This internalin family of proteins has proven roles in virulence and host-pathogen interactions (38). Studies by Favaro et al. (12) and Rossi et al. (19) reported the nonpathogenic *Listeria* spp. Virulent strains, which caused infection in humans comprised of *inl*A, *inl*B, and incomplete *LIPI*-1 consisting of *prf*A, *hly*, and *plc*A genes. However, this was not the case in the present study as none of the genomes possess these genes. The present study conducted a complete genome analysis suggesting the presumed nonpathogenic *L. innocua* strains, especially ST132 and ST637, have more virulence factors and resistance genes than *L. welshimeri* strains isolated in SA. The presence of identical virulence and resistance genes could indicate horizontal gene transfer between the presumed nonpathogenic and pathogenic *Listeria* strains in SA.

Mobile genetic elements typically give rise to a diverse functional variation in the *Listeria* accessory genome (6, 39). Blasting of nucleotide sequences of the identified plasmids against the NCBI database revealed homology of these plasmids at more than 95% similarity with the virulence plasmid J1776 and *pLGUG1*. These plasmids are known to encode a number of genetic markers related to stress resistance (6). Some studies have shown L. monocytogenes can acquire resistance genes from the environment through plasmids and transposons, leading to the gradual increase in L. Monocytogenes resistance (6, 40). Wu et al. (28) showed *Listeria* carry multiple resistance genes *tetA*, *tetM*, *ermA*, *ermB*, *ermC*, and *aac(6′)-Ib*. Generally, resistant genes *tetB* and *tetM* are frequently detected in mobile plasmids (41). This included features related to disinfectant resistance (*bcrABC* and *Tn6188_qac_1*), LGI3, and other stress resistance markers. The J1776 is a common virulent plasmid found in L. monocytogenes (26, 42). The *pLGUG1* specific genes encode a MATE family multidrug efflux pump in *listeria* spp. (40, 43). The present study revealed this was also common in *L. innocua* ST1085, ST132, and ST637 in the present study. Furthermore, the most common prophages detected were PHAGE_Lister_vB_LmoS_188 and PHAGE_Lister_LP_030_3 in *L. innocua* ST537. However, such prophages are primarily common in L. monocytogenes with adaptation and evolution roles in distant and closely related strains (23, 26, 44). Similarly, Palma et al. (23) and Orsi et al. (44) reported the differences in a prophage sequence differentiate four similar genome backbone L. monocytogenes isolates, indicating the importance of prophages in the differentiation of closely related L. monocytogenes. For example, the role of prophages in the adaptation and evolution of bacterial spp. has been reported to be associated with the acquisition of a prophage that contained a unique combination of virulence genes, which was probably generated through several recombination events (44).

Previous studies have successfully detected CRISPR repeats in L. monocytogenes genomes (3, 43, 45, 46). The CRISPR-Cas systems detected by previous studies were primarily type IB and IIA which is in agreement with the present work that primarily detected type IB and IIA in *Listeria* spp. Several pieces of evidence suggest at least some of the CRISPR-Cas systems detected in L. monocytogenes are functional with spacers that exactly match sequences of known *Listeria* phages and plasmids (45). The presence of CRISPR-Cas systems in the current *Listeria* genomes may suggest the lack of mobile genetic elements could be the result of a functional CRISPR-Cas systems in these isolates. The presence of CRISPR-Cas systems in many *Listeria* genomes suggests their suitability for biotechnological application against L. monocytogenes, although correct selection and adaptation of the systems will be crucial (45).

### Conclusion.

In order to gain an improved understanding of genome evolution in the genus *Listeria*, with particular attention to the evolution of virulence and stress tolerance, genome sequencing was conducted on isolates recovered from food and FPE. Analysis of 43 genome sequences representing two *Listeria* spp. (*L. innocua* and *L. welshimeri*) point to the fact that the presumed nonpathogenic *Listeria* spp. is unlikely to cause disease manifestation compared with pathogenic spp. due to the low occurrence of virulence factors such as *inl*A, *inl*B, and *LIPI*-1. The presence of genetic loci that have been previously associated with adaption/survival in stressful conditions was high in *L. innocua* especially *L. innocua* ST132 than in any of the *L. welshimeri* strains. The study highlights the low occurrence of important core genes could be the result of a functional CRISPR-cas system in the *Listeria* genomes.

## MATERIALS AND METHODS

### Genome assembly and annotation of the bacterial strains.

The isolates used in this study were collected between 2014 and 2019 and submitted at Onderstepoort Veterinary Research, SA, as part of research and/or routine diagnostics services. A total of 258 isolates from different geographical locations (processing facilities, butcheries, abattoirs, retail outlets, and cold stores) in SA were selected for sequencing. Of these isolates, 41 of these isolates were presumed to be nonpathogenic strains (*L. innocua*, 38 isolates and *L. welshimeri*, 3 isolates), and 217 isolates were pathogenic strains (L. monocytogenes). The L. monocytogenes results have been previously published by Mafina et al. (26) and Matle et al. (27). Here, we present the presumed nonpathogenic strains and three L. monocytogenes reference genomes which were used in this study for comparison purposes (Table 1). The presumed nonpathogenic isolates were preserved as lyophilized and subsequently revived by inoculation into brain heart infusion (BHI) broth and incubated at 37°C for 18 to 24 h. According to manufacture instructions, genomic DNA from BHI broth culture was extracted using the High Pure PCR Template Preparation Kit (Roche, Potsdam, Germany) and the quality of the DNA was assessed using Qubit flourimetric quantitation (Thermo Fischer Scientific, Waltham, MA, USA). The DNA libraries were prepared using Truseq DNA library preparation kit (Illumina, San Diego, CA, USA) following the manufacturer’s instructions. The prepared libraries were loaded for 2 × 300 bp reads sequencing on Illumina HiSeq and Miseq Sequencing platform (Illumina, San Diego, CA, USA). The raw read quality was assessed with FastQC v.0.11.9 (47), and the adapters and low-quality reads were trimmed using Trimmomatic v.0.39 default settings (48). SPAdes v.3.13.1 program (49) was used for *de novo* assembly of each isolate. The resulting genome assembly was further quality assessed with QUAST v.5.0.2 (50) and annotated using Prokka v.1.13.7 (51).

### Multilocus sequence type, pan-genome, and phylogenetic construction.

Multilocus sequence type (MLST) profiles were obtained from the *Listeria* database hosted by the Pasteur Institute, France (http://bigsdb.pasteur.fr/listeria/). The MLST v.2.18.0 (52) was used to align reads against these profiles to determine the sequence types (STs) of each isolate.

The pangenome composition was extracted using Roary (53) and a core genome phylogenetic tree constructed with IQ-TREE v.1.6.6 (54). Pan-genome clusters were defined as core-genes: present in all isolates; soft-core genes: present in at least 95% of isolates; shell-genes: present between 15% and 95% of isolates; and cloud-genes: present in less than 15% of isolates. The core genome phylogenetic tree constructed using IQ-TREE was visualized using iTOL v.6.5 (55).

### Genome screening for resistance markers and virulence factors.

The resistance genes, *Listeria* genomic islands, and virulence factors were searched against a database created with genes retrieved from the *Listeria* database hosted by the Pasteur Institute, Paris, France (http://bigsdb.pasteur.fr/Listeria/). The plasmids database (PLSDB) (56) was searched for complete bacterial plasmid sequences. To identify putative prophages, genome assemblies were searched by the PHASTER (PHAge Search Tool – Enhanced Release) server (57). CRISPRCasFinder web server v1.1.2 (24) was used to search for CRISPR-Cas genes in the study genomes. All the genes of interest were interrogated using ABRicate v0.8.10 (https://github.com/tseemann/ABRicate) with minimum identity and coverage cut-offs values set by default settings.

### Ethical statement.

Ethical approval was obtained from the University of Pretoria, Faculty of Natural and Agricultural Sciences Research Ethics Committee (NAS324/2020). All methods in this study were approved by the University of Pretoria, Faculty of Natural and Agricultural Sciences Research Ethics Committee, and performed in accordance with the relevant guidelines and regulations.

### Data availability.

The data sets generated during and/or analyzed during the current study are available in the NCBI Sequence Read Archive (SRA) repository, BioProject ID accession number PRJNA804318 and the draft genomes are available at BioProject ID accession number PRJNA863749.
